# Review and Chemoinformatic Analysis of Ferroptosis Modulators with a Focus on Natural Plant Products

**DOI:** 10.3390/molecules28020475

**Published:** 2023-01-04

**Authors:** Višnja Stepanić, Marta Kučerová-Chlupáčová

**Affiliations:** 1 Laboratory for Machine Learning and Knowledge Representation, Ruđer Bošković Institute, Bijenička 54, 10000 Zagreb, Croatia; 2 Department of Pharmaceutical Chemistry and Pharmaceutical Analysis, Faculty of Pharmacy in Hradec Králové, Charles University, Ak. Heyrovského 1203/8, 500 05 Hradec Králové, Czech Republic

**Keywords:** ferroptosis, inducers, inhibitors, drug-likeness, cancer, neurodegenerative, polyphenol

## Abstract

Ferroptosis is a regular cell death pathway that has been proposed as a suitable therapeutic target in cancer and neurodegenerative diseases. Since its definition in 2012, a few hundred ferroptosis modulators have been reported. Based on a literature search, we collected a set of diverse ferroptosis modulators and analyzed them in terms of their structural features and physicochemical and drug-likeness properties. Ferroptosis modulators are mostly natural products or semisynthetic derivatives. In this review, we focused on the abundant subgroup of polyphenolic modulators, primarily phenylpropanoids. Many natural polyphenolic antioxidants have antiferroptotic activities acting through at least one of the following effects: ROS scavenging and/or iron chelation activities, increased GPX4 and NRF2 expression, and LOX inhibition. Some polyphenols are described as ferroptosis inducers acting through the generation of ROS, intracellular accumulation of iron (II), or the inhibition of GPX4. However, some molecules have a dual mode of action depending on the cell type (cancer versus neural cells) and the (micro)environment. The latter enables their successful use (e.g., apigenin, resveratrol, curcumin, and EGCG) in rationally designed, multifunctional nanoparticles that selectively target cancer cells through ferroptosis induction.

## 1. Introduction

Cancer is one of the most common causes of death in humans worldwide [1]. Prevention programs and early cancer detection through regular medical check-ups and with the use of specific biomarkers, as well as the development of novel therapeutics (such as the development of various protein kinase inhibitors and immunotherapeutics), reduce cancer mortality [2,3]. Cancer therapy (chemotherapy, targeted therapy, radiotherapy, or immunotherapy) generally aims to destroy cancer cells without too many harmful effects on healthy cells. The induction of natural, programmed cell death pathways through the use of low-molecular-weight (MW) compounds has been widely explored as a way to combat death-escaping cancer cells. In cancer chemoprevention and chemotherapy, the induction and promotion of cancer cell apoptosis by small molecule agents have been extensively studied [4,5]. However, the main limitation of this approach is that cancer acquires resistance to such drugs, including targeted therapies, leading to their failure [4]. Dysregulated mechanisms that sustain cancer resistance to various other types of cell death pathways have also been studied in solid tumors and hematological malignancies [6].

Cancer initiation and promotion are generally linked with oxidative stress [7]. Oxidative stress causes DNA mutations, cell component damage, and pro-oncogenic signaling and, thus, triggers and sustains carcinogenesis [8]. Sustained overproduction of reactive oxygen species (ROS) may lead to persistent, chronic oxidative stress and injury through nonlethal modifications of normal cellular growth control mechanisms such as modified intercellular communication, protein kinase activity, membrane structure and function, and gene expression. Most conventional chemotherapeutic agents increase ROS production and, thus, cause cell and tissue damage and activate an inflammatory response.

Ferroptosis is a regulated cell death mechanism caused directly by (over)production and accumulation of specific kinds of ROS, which may enable the selective killing of cancer cells without causing significant toxicity to normal cells. The druggability of ferroptosis by low-MW compounds has already been demonstrated. In fact, ferroptosis was discovered to be a programmed cell death pathway by using low-MW compounds [9]. The treatment of an NRAS oncogene mutant containing HT-1080 fibrosarcoma cells with the compound erastin (10 μM) induced a time-dependent, continuous increase in cytosolic and lipid ROS, which resulted in cell death with a distinct non-apoptotic phenotype. Cell death was suppressed by each of the following low-MW agents: iron chelators deferoxamine (100 μM) and ciclopirox olamine (5 μM), the glutathione peroxidase mimetic organoselenium compound ebselen (5 μM), the mitogen-activated protein kinase (MEK) inhibitor U0126 (5 μM), and the antioxidants trolox (100 μM) and ferrostatin-1 (EC_50_ = 60 nM). Since ferroptosis was described as a distinct regulated cell death pathway in 2012, more than a few hundred low-MW inducers and inhibitors of ferroptosis have been reported.

Recently, many reviews on ferroptosis relating to various biological aspects have been published [10,11,12]. Herein, we collect sets of 30 representative inducers (Table 1 and Table S1) and 48 suppressors/inhibitors (Table 2 and Table S2) of ferroptotic cell death with a MW of less than 800 and analyze them in relation to structural, physicochemical/drug-likeness, and biological/pharmacological aspects. Thereafter, the review focuses on describing the biological activities/effects of the subset of (poly)phenolic ferroptosis modulators since, to our knowledge, there has been no such comprehensive review of polyphenols as ferroptosis modulators [13,14,15,16,17]. We focus only on the activities of (poly)phenolic compounds (many of which are already known) in conjunction with their reported (anti)ferroptotic effects. We review their influence on ferroptosis through activities affecting the three major components of ferroptosis. Table 1 and Table 2 list these activities for natural plant molecules and put them in the context with other ferroptosis modulators with analogous effects.

## 2. Ferroptosis—Three Main Factors

Ferroptosis was described in 2012 as a non-apoptotic cell death mechanism. It is a regulated form of cell death that is triggered and driven by the (over)production and accumulation of lipid and phospholipid (hydroxy)peroxides, the formation of which is specifically mediated by Fe^2+^ ferrous ions. It is named after Fe^2+^ ferrous ions since cytosolic and mitochondrial Fe^2+^ ions are essential factors in ferroptosis. Once ignited, ferroptosis can remain local or rapidly spread to surrounding cells, depending on the ignition mechanism [19]. It is morphologically, biochemically, and genetically distinct from apoptosis, necrosis, and autophagy but similar to oxytosis. Ferroptosis is closely associated with mitochondria, and its primary morphological markers are aberrant mitochondria characterized by a reduced number of mitochondrial cristae, inner membrane condensation, outer membrane rupture, and size shrinkage [20,21]. Physiologically, ferroptosis may contribute to embryonic development, erythropoiesis, aging, and antiviral and anticancer defense mechanisms [11]. Pathologically, it is associated with neurological diseases, myocardial infarction, atherosclerosis, renal and liver diseases, and cancer.

Three factors are necessary for the induction of and maintaining ferroptosis. These are: (i) the intracellular accumulation of Fe^2+^ ferrous ions, (ii) the accumulation of lipid peroxides generated from polyunsaturated fatty acids (PUFA) with bis-allylic fragments localized mainly in membranes, and (iii) the deficient repair of lipid peroxides. It is possible to induce or inhibit ferroptotic cell death in a relatively straightforward manner by directly or indirectly targeting one of the three necessary factors. They are considered markers for ferroptosis.

Iron is an essential metal in the human body, and its uptake, distribution, storage, and retrieval are coordinated at cellular and systemic levels by a complex and finely balanced network of regulatory pathways. Iron is involved in a variety of physiological functions and processes, including DNA replication, the tricarboxylic acid cycle, ATP production via the electron transport chain, and signal transduction. It is a cofactor in 6.5% of all human enzymes, localized mainly in the endoplasmic reticulum and mitochondria in the form of iron ions, heme, or iron–sulfur (FeS) clusters [22]. Its aberrant metabolism, leading to excessive Fenton reactions and/or impairment of mitochondrial function and energy metabolism, induces ferroptosis. Free, non-protein-bound Fe^2+^ ions can be quickly released from labile iron pools that are available within living cells and serve as a transient hub of the cellular iron metabolism. Intracellular accumulation of free iron can lead to high production of ROS which can override the antioxidant defense of a cell.

Other forms of regulated cell death mechanisms such as apoptosis, necroptosis, and pyroptosis may also depend on iron-induced ROS and oxidative stress. However, ferroptosis is dependent on lipid peroxidation. Ferroptosis is characterized by excessive peroxidation of PUFA bound in certain phospholipids such as phosphatidylethanolamines (e.g., peroxides of phosphoethanolamines (PEs) with arachidonic acid (AA) (18:0/20:4(5*Z*,8*Z*,11*Z*,14*Z*) PE-AA) or adrenic acid (AdA) (18:0/22:4 (7*Z*,10*Z*,13*Z*,16*Z*) PE-AdA), phosphatidylcholines, and other types of phospholipids [23]. These phospholipids are synthesized mainly in the membranes of mitochondria and the endoplasmic reticulum. Lipids are peroxidized through non-enzymatic and enzymatic mechanisms involving Fe^2+^ ions. In the non-enzymatic Fenton reaction of Fe^2+^ ions with hydrogen peroxide (H_2_O_2_), hydroxyl radicals (HO•) are produced, which can cause oxidative damage to cellular components such as lipids and induce cell death. Lipoxygenase (LOX) enzymes are non-heme, iron-containing dioxygenases that catalyze the stereospecific oxygenation of free and esterified PUFAs, generating a spectrum of bioactive lipid mediators that can initiate autocatalytic lipid autoxidation.

Since lipid peroxidation fuels the spread of ferroptosis, regulation of the activity and expression of proteins involved in lipid metabolic pathways has a major impact on ferroptosis [24]. These are the enzymes responsible for the formation of phospholipids and their incorporation into various cell membranes (ACSL4 (acyl-CoA synthetase long-chain family member 4/long-chain fatty-acid-CoA ligase 4) and LPCAT3 (lysophospholipid acyltransferase 5)), as well as enzymes regulating lipid peroxidation such as LOXs and GPX4 (glutathione peroxidase 4). Lipid peroxide radicals are neutralized non-enzymatically by exogenous, lipophilic free radical scavengers such as α-tocopherol and β-carotene and enzymatically by the selenoprotein GPX4. GPX4 is unique among the eight GPX isoforms in that it is the only enzyme capable of reducing oxidized, esterified fatty acids and cholesterol hydroperoxides, thus, protecting against lipid peroxidation in cells and structural function in mature sperm cells [25,26]. It reduces fatty acids/phospholipid hydroperoxides to lipid alcohols with the help of glutathione (GSH) as a cofactor, even if they are incorporated in lipoproteins and membranes. There are three isoforms of GPX4: mGPX4 distributed in mitochondria, nGPX4 distributed in nucleoli, and cGPX4 distributed in the nucleus and cytosol and also strongly associated with membranes. GPX4 activity decreases under GSH depletion and can also be directly inhibited via a covalent interaction with selenocysteine in the active site. Deficiency of GPX4 in quantity and/or activity leads to ferroptosis. Its cofactor, GSH, is synthesized from cysteine and glutamate, and cystine is a precursor of cysteine. GPX4 activity can be reduced by depletion of GSH through inhibition of the cystine/glutamate antiporter X_c_^‾^ system, which exchanges intracellular L-glutamate for extracellular L-cystine across the cellular plasma membrane. The X_c_^‾^/GSH/GPX4 axis crucially controls ferroptosis. The heterodimeric X_c_^‾^ system consists of two transmembrane amino acid transporters: SLC3A2 (solute carrier family 3 member 2) and cystine/glutamate transporter SLC7A11 (solute carrier family 7 member 11). Glutamate itself can be replenished by importing glutamine via transporter SLC1A5. Cystine is reduced inside cells to cysteine. The enzyme ACSL4 enriches cellular membranes with long ω-6 PUFAs, determining the ferroptosis sensitivity of cells [27]. It was found to be preferentially expressed in a panel of basal-like breast cancer cell lines.

## 3. Ferroptosis as a Potential Therapeutical Target

Cell death is impaired in cancer, and the common method of cancer therapy is to induce death mechanisms in immortal cancer cells. Cancer cells are characterized by a rapid proliferation rate that requires a high iron load, and they are well adapted to acquire iron and prevent its loss [28]. Iron plays an important role in modulating the tumor microenvironment and metastasis, maintaining genomic stability, and controlling epigenetics. To meet the high iron demand, neoplastic cells remodel iron metabolic pathways, including iron uptake, storage, and efflux, making the manipulation of iron homeostasis an important approach for cancer therapy [29]. Metabolic reprogramming of cancer cells also involves mitochondrial dysfunction and dysregulated p53 expression, which has been implicated in the regulation of ferroptosis [30,31]. To facilitate iron uptake, TfR1 (transfer iron protein receptor 1) is highly expressed on the surface of cancer cells, and iron is accumulated within cells transformed with the oncogene RAS because of the upregulation of TfR1. Ferritin, an intracellular protein that stores iron, is also elevated in many cancers, including breast cancer, and can be used as a prognostic marker for breast cancer progression.

The process of ferroptosis involves signaling pathways that play a role in cancer biology, such as the AMP-activated protein kinase (AMPK)-RAS/MAPK and AMPK/mTOR/p70S6k pathways and the NRF2-KEAP1 pathway. NRF2 (nuclear factor erythroid 2-related factor 2) is an important transcription factor in the regulation of oxidative stress and plays a major role in the induction of drug insensitivity or resistance in cancer cells. Its activity affects the expression of antiferroptosis genes encoding for GPX4, SLC7A11, and iron storage protein ferritin subunits—FTH1 (ferritin heavy chain 1) and FTL (ferritin light chain) [32]. Cancer chemoresistance may be caused by activation of the NRF2 and downstream NRF2/FTH1 or NRF2/SLC7A11 pathways, resulting in the lowering of free intracellular iron (TfR1 downregulation and ferritin upregulation) or enhanced neutralization of lipid peroxides (GPX4 and FSP1 (ferroptosis suppressor protein 1) upregulation) compared to those in drug-sensitive cells. Thus, ferroptosis is a specific weakness of cancer suitable for use in the treatment of certain therapy-resistant cancers. Inducing ferroptosis in synergy with classic chemotherapeutic agents can sensitize cancer cells to treatments.

Several dozen compounds have been reported to induce ferroptosis in cancer cells by direct modulation of the ferroptosis targets (Table 1). Sensitivity profiling of 177 cancer cell lines to 12 ferroptosis-inducing small molecules (including erastin, RSL3, and their analogs) revealed that diffuse large B-cell lymphomas and renal cell carcinomas are particularly susceptible to ferroptosis through GPX4 inhibition [26]. Some natural inducers promote ferroptosis by regulating the ROS/AMPK/mTOR signaling pathways to inhibit cancer cell viability and proliferation, such as dihydroartemisinin (DHA) and amentoflavone.

Since ferroptosis is associated with neurodegeneration, numerous modulators have been discovered through studies of neurological, pathological conditions. It was found that ferroptosis induced by erastin is similar in cancer cells and primary neurons [33]. Therefore, we collected compounds discovered to act as ferroptosis modulators in cancer and/or neuronal cells and analyzed them in relation to chemical/structural and physicochemical/drug-likeness molecular aspects.

**Figure 1:**
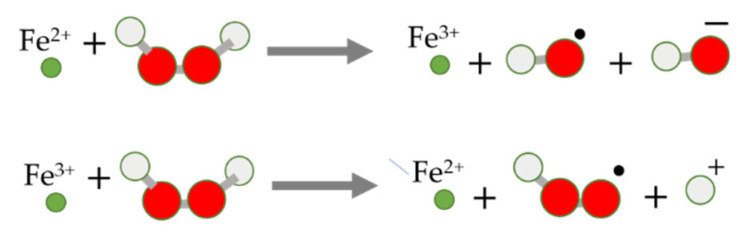
Fenton reaction of iron ions and H_2_O_2_ for production of powerful ROS. Under the condition of accumulated reactants, it can become uncontrolled, causing extensive oxidative damage and death.

In the context of ferroptosis, it should be stressed that an additional, important common factor in cancer, neuronal networks, and ferroptosis is hydrogen peroxide (H_2_O_2_) (Figure 1). Cancer cells including melanomas, neuroblastoma, colon carcinoma, ovarian carcinoma, and cancer-associated fibroblasts, as well as myofibroblasts, macrophages, and neutrophils, are the major producers of H_2_O_2_ [34]. H_2_O_2_ is not only used as an effective biological weapon but is also an important signaling molecule in cancer and neuronal networks [35,36]. The localized and concentrated production of H_2_O_2_ and ROS is enabled by the packaging of active ingredients including ferroptotic modulators in multifunctional nanosystems.

The iron-seeking phenotype of cancers can be exploited in two ways: (i) to restrict iron availability and (ii) to exploit the redox properties of excess iron to promote cytotoxic oxidative stress in cancer cells. To foster ROS generation, nanoparticles provide rationally designed strategies to preferentially deliver drugs/active ingredients into cancer cells [37]. Recently, different ferroptotic inducers have also been applied in the form of rationally designed, multifunctional nanoparticles on a variety of cancer cells. Their efficacy has been enhanced by the delivery of nanoparticles to a target place in chemotherapy, radiotherapy, and immunotherapy [38]. The development of nanotherapeutics offers a route to overcome the toxic, off-target effects of ferroptosis on normal cells and the shortcomings of ferroptosis-driven therapeutics due to their low bioavailability caused by their low aqueous solubility and membrane permeability. Some ferroptosis-driven nanotherapeutics contain the two basic elements of the Fenton response: Fe^2+^ ions and/or H_2_O_2_ to trigger and promote a Fenton reaction in cancer cells. The lower pH caused by hypoxia in the tumor microenvironment facilitates the release of iron ions from nanomaterials, which triggers the Fenton reaction and leads to the ferroptotic death of cancer cells. Some nanotherapeutics enhance the uptake of ferroptosis inducers into cancer cells, while others provide exogenous regulation of lipid peroxidation to cancer cells through, e.g., PUFA supplementation.

## 4. Chemical Aspects of Ferroptosis Modulators

The induction and inhibition of ferroptosis by small compounds have been well characterized so far. They alter the concentration of ROS and, thus, of (per)oxidized lipid species through general mechanisms such as free radical scavenging and iron chelation and/or by modulation of specific biochemical pathways.

Ferroptosis inducers are compounds that stimulate iron accumulation and/or inhibit GPX4 expression and/or activity, thereby promoting lipid ROS production and accumulation. GPX4-regulated ferroptosis can be induced in two ways. Class I inducers such as erastin inhibit GPX4 by causing depletion of its cofactor, intracellular GSH. Class II inducers such as RSL3 directly inhibit GPX4 by binding to it. Ferroptosis inhibitors, such as iron chelators and lipophilic antioxidants, have the opposite effect of reducing lipid ROS concentration.

**Figure 2:**
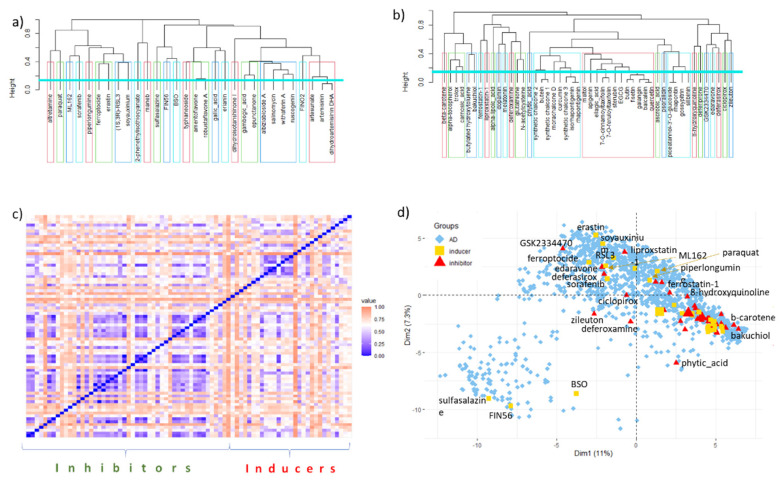
Analysis of structural diversity of ferroptosis modulators described with MACCS fingerprints and using the Jaccard index (1 − TC) as a dissimilarity measure (*y*-axis). Cluster dendrograms for (**a**) 30 inducers and (**b**) 48 inhibitors. Clusters are denoted with borders drawn at a TC level of 0.6, while the cyan line corresponds to a TC of 0.85. (**c**) Structural (dis)similarity of ferroptosis modulators. More blue/red values denote greater/less structural similarity. (**d**) The ferroptosis inducers and inhibitors fall within the chemical space of approved drugs (AD) which is represented by the first two PCA components, explaining 18.3% of the variance in MACCS fingerprints.

In this analysis, a set of 78 organic ferroptosis modulators (Table 1 and Table 2) with a MW up to 800 is described and compared in relation to the aspects of structural diversity and drug-likeness. The collected ferroptosis modulators represent different chemical scaffolds that have been employed as privileged scaffolds for designing novel chemical libraries of ferroptosis inducers or inhibitors and for structure–activity relationship (SAR) analysis. The dendrograms in Figure 2a,b depict the structural diversity of the collected inducers and inhibitors, respectively. The collected set of ferroptosis inducers is a group of chemically diverse compounds. Employing a TC value of 0.85 (Jaccard = 1 − TC of 0.15; cyan line in Figure 2a,b) as a lower limit of chemical similarity, only artemisinin and its dihydrogenated derivate DHA are mutually structurally similar, and they are placed within the same group. Other inducers are structurally unique compounds forming one-member clusters. In comparison, ferroptosis inhibitors are more mutually similar molecules (Figure 2b). The 23 polyphenolic antioxidants are grouped into three clusters as they possess the same core scaffolds with various numbers and positions of OH substituents and/or sugars. The inducers and the inhibitors have a few structural similarities (Figure 2c).

The structures of ferroptosis modulators are compared to those of drugs available on the market(s) by principal component analysis (PCA). Most of the modulators (53/78 in total; 16/30 inducers; 37/48 inhibitors) are grouped in the bottom-right part of the PCA score plot (Figure 2d), following their proximity already shown in the dendrograms (Figure 2a,b). They are inhibitors with antioxidative (20/25), iron chelation (11/15), and GPX4 activation (3/3) modes of action plus inducers with iron accumulation (6/6) or GPX deactivation (5/10) effects. They are clustered together with around two-thirds of the drugs from the ATC groups D (dermatologicals), G (genito-urinary system and sex hormones), and H (systemic hormonal preparations, excluding sex hormones and insulins). The rest of the ferroptosis modulators are scattered in the score plot.

The placement of ferroptosis modulators within the structural space of drugs also implies their drug-like character (Figure 2d). The general, whole-molecule physicochemical properties of compounds are known to influence their ADME (absorption, distribution, metabolism, and excretion) profile and drug-likeness [39]. Although research on the significance of ferroptosis in anticancer therapy has progressed, there are still limitations to its clinical application because of the low solubility and poor membrane permeability of ferroptosis modulators (Tables S1 and S2). For instance, the iron-chelating agent deferoxamine is a classic ferroptosis inhibitor used to avoid excessive ferroptosis and injury to normal cells and tissues. However, its low solubility limits its application. The poor ADME profiles of compounds not only decrease target efficiency but also increase the likelihood of undesirable, off-target effects on normal cells.

**Figure 3:**
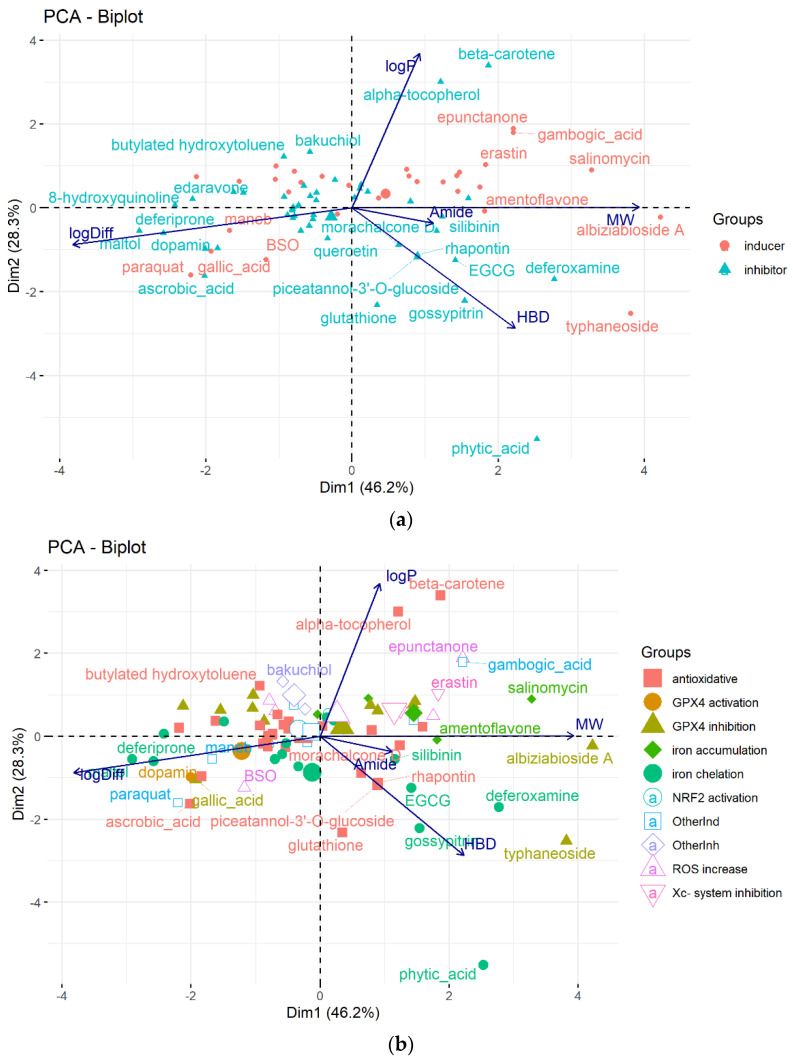
The PCA biplot generated in terms of molecular traits (blue arrows) that describe similarity/difference in the physicochemical space of 78 ferroptosis modulators colored according to (**a**) the class of inhibitors/inducers and (**b**) the mode of action.

The majority of low-MW ferroptosis modulators are natural or natural-based molecules that fit the drug-likeness profile in terms of log*P* values and numbers of H-bond donors, which are considered the two most important parameters in predicting oral bioavailability and drug-likeness [39]. The 10 ferroptosis modulators do not obey the well-known Lipinski rule of five, mainly due to molecular size and the number of H-bond donors greater than 500 and 5, respectively. The 75 molecular physicochemical features important for biological activities were calculated and used for uncovering the (di)similarity of ferroptosis modulators by applying PCA. Five statistically significant molecular features were found to be important for the differentiation of ferroptosis modulators in a physicochemical space. They are: molecular weight (MW), diffusion coefficient in water (Hayduk–Laudie formula) (log(Diff × 10^−5^/(cm^2^/s)), the number of hydrogen-bond-donating atoms (HBD), lipophilicity coefficient (log*P*), and the number of amide groups (Amide) (Figure 3). These descriptors are known to influence aqueous solubility and distribution properties such as passing biological membranes [40].

Ferroptosis modulators with different modes of action have different physicochemical profiles (Figure 3b). Ferroptosis inhibitors with antioxidant activity are diverse molecules in terms of their structures and mechanisms of action, as well as their physicochemical profiles. Small, hydrophilic antioxidants such as vitamin C, *N*-acetylcysteine, and GSH acting in the aqueous environment are placed in the lower-left part of the PCA plot. Relatively large lipophilic antioxidants such as α-tocopherol and β-carotene scavenge lipid ROS in the hydrophobic environment in membranes. Iron-chelating inhibitors are scattered in the PCA plot, which illustrates that they have various H-bond/H-atom-donating capacities and molecular size, but almost all lie under the *x*-axis, showing that they are, in general, hydrophilic compounds. X_c_^‾^ system inhibitors and iron accumulation inducers are among the largest and most lipophilic ferroptosis modulators.

The ferroptosis-activating or -suppressing effects of low-MW compounds were mainly detected in terms of their in vitro anticancer and neuroprotective effects on cancer cell lines and neuronal cells, respectively. Hence, the estimation of the capacity of a compound for crossing the BBB and retention in the brain once taken up (Table 1, Table 2, Tables S1 and S2) was used to assess whether or not a compound is likely to be active in the central nervous system (CNS). CNS activity may be a(n) (un)desirable side effect of a cancer chemotherapeutic. Dozens of inducers (e.g., FINO2, dihydroisotanshinone I, artemisinin, DHA, 2-phenethylisothiocyanate, altretamine, piperlongumine) and inhibitors (e.g., α-tocopherol, bakuchiol, butylated hydroxytoluene, liproxstatin-1, ferrostatin-1, 8-hydroxyquinoline) are estimated to cross the BBB and be retained in the brain (Tables S1 and S2) [33,41].

## 5. Biological Aspects of Ferroptosis Modulators

We further analyzed the SAR for ferroptosis modulators. We used the ClassyFire approach to assign chemical (super)classes (Tables S1 and S2) [42]. The class was determined by the core scaffold in a compound. Most ferroptosis modulators are natural products or semisynthetic derivatives belonging to the superclasses of “phenylpropanoids and polyketides” (29 compounds) or “lipids and lipid-like molecules” (13 compounds) according to their biosynthetic origin (Table 1 and Table 2). The majority of the remaining modulators are derivatives of organic acids or compounds with structurally diverse, heterocyclic scaffolds.

Almost one-third of the inducers and some inhibitors belonging to the “lipids and lipid-like molecules” are prenol lipids synthesized by the condensation of isoprene subunits. This class includes the inducers artesunate, ruscogenin, and salinomycin, which increase intracellular iron(II) concentration and lipid peroxidation, and lipophilic antioxidant inhibitors α-tocopherol and β-carotene. Of the 29 ferroptosis modulators from “phenylpropanoids and polyketides”, six molecules are ferroptosis inducers, and most of the remaining 23 molecules are polyphenolic ferroptosis inhibitors belonging to the flavonoid stilbene and linear 1,3-diarylpropanoid chalcone types. Other ferroptosis inhibitors are lipophilic (vitamin A and α-lipoic acid) and hydrophilic (GSH, vitamin C, dopamine) compounds (Table 2). In this article, we focus mainly on (poly)phenols that act as ferroptosis inducers and inhibitors, or both, and which belong mainly to the superclass of “phenylpropanoids and polyketides”.

### 5.1. Ferroptosis Inducers

The first ferroptosis modulators discovered were the inducers erastin and RAS-selective lethal 3 RSL3 ((1*S*,3*R*)-RSL3 given the stereochemistry). Erastin is an inhibitor of the X_c_^‾^ system, while RSL3 is a covalent inhibitor of GPX4. These synthetic compounds were discovered as ferroptosis inducers by the screening of diverse compounds. In 2003, erastin was detected to induce a new form of non-apoptotic cell death in RAS-overexpressing cancer cells [43]. In 2007, it was found to bind directly to voltage-dependent anion channel protein VDAC2 in the mitochondrial outer membrane, leading to mitochondrial production of ROS, mitochondrial dysfunction, and non-apoptotic cell death of RAS-overexpressing cancer cells. In 2008, RSL3 was revealed to induce cell death by involving labile iron [44]. Erastin, glutamate, sorafenib, and sulfasalazine are type I inducers that block the X_c_^‾^ system, resulting in a decrease in GSH levels. RSL3, altretamine, ML162, and FIN56 are type II inducers that affect GPX4 either by directly inhibiting the enzyme or by reducing its expression level. Erastin and RSL3 are not readily bioavailable molecules. However, erastin analogs (e.g., PRLX 93936) and other X_c_^‾^ system inhibitors, such as multikinase inhibitors sorafenib and sulfasalazine, as well as GPX4 inhibitors, e.g., altretamine and withaferin A, are under clinical anticancer investigation [45].

Some currently used drugs have been found to induce ferroptosis in addition to their already known mechanisms of action [46]. For example, the cancer chemotherapeutic agent cisplatin not only prevents DNA repair but also induces ferroptosis by depleting intracellular GSH in lung A549 and colon HCT116 cancer cells [47]. The most potent anti-malarial drug isolated from traditional Chinese herb *Artemisia annua* L., artemisinin, and its derivative DHA, selectively targets KRAS-reprogrammed pancreatic cancer cells that are resistant to apoptosis. They accumulate in lysosomes and increase ferritin degradation and, thus, iron release, which promotes the accumulation of cellular ROS and leads to ferroptotic cancer cell death. DHA was found to accelerate the degradation of ferritin through downregulation of the activity of the mTOR/p70S6k signaling pathway by activating the phosphorylation of AMPK. DHA also induced ferroptosis of acute myeloid leukemia (AML) cells through autophagy by regulating the activity of the AMPK/mTOR/p70S6k signaling pathway. The water-soluble artemisinin derivative artesunate selectively killed head and neck cancer (HNC) cells but not normal cells by decreasing cellular GSH level and increasing lipid ROS levels [48]. Inhibition of the NRF2 pathway (by NRF2 genetic silencing or by trigonelline) increased the artesunate sensitivity of ferroptosis-resistant HNC cells.

Polyphenols are natural products known for a plethora of bioactivities, including their diverse effects on ferroptosis cell death [13,14,15,16,17,49]. They have shown significant anticancer activity in vitro, even against an aggressive type of cancer, and their chemopreventive role has been summarized elsewhere [50,51]. Grounded in many in vitro and in vivo (mostly rodent) preclinical studies, clinical trials with polyphenols alone or in a combination with anticancer drugs have been carried out on hormone-dependent prostate and breast cancers, bladder and renal cancers, and colorectal and lung cancers, as well as on leukemia. They can trigger several cell death mechanisms simultaneously. They can act as ferroptosis inducers or inhibitors or both. It is known that the dominant target activity of polyphenols depends on the dose, treatment duration, and cell/tissue specificity [52].

Polyphenols have already been used in dermatology and are known to exert antiproliferative, proapoptotic, and antimetastatic activities on melanoma cell lines, whereas they have no cytotoxic effect in healthy cells [53,54]. Since ferroptotic pathways contribute to the regulation of the differentiation state of melanoma cells and their resistance to certain therapeutic agents, ferroptosis inducers are expected to have strong therapeutic potential in melanoma [55]. The majority of collected polyphenolic ferroptosis modulator representatives were found to be structurally most similar to drugs from related ATC groups D, G, and H (Figure 2d). The local, topical application of polyphenols may have advantages over their oral administration because these molecules are generally rapidly metabolized and weakly bioavailable, in addition to having multitargeting effects. The observed effects on cancer cells of ferroptosis inducers from the “phenylpropanoids and polyketides” superclasses (except gallic acid) are described further.

Typhaneoside, a major flavonoid in the extract of *Typha* spp. (Typhaceae) pollen, was found to strongly inhibit the proliferation and growth of AML cells by interacting with multiple targets simultaneously [56]. Typhaneoside (40 µM) induced apoptosis, autophagy, and ferroptosis in AML cells. The induction of ferroptosis was iron-dependent and attended by mitochondrial dysfunction and reduced GSH and GPX4 levels. It triggered autophagy in AML cells by promoting AMPK signaling, which contributes to ROS accumulation, ferritin degradation, and ferroptotic cell death. The anticancer activity of typhaneoside was also confirmed in vivo using BALB/c mice xenografts bearing HL60 cells.

A biflavonoid obtained by the oxidative coupling of two molecules of apigenin robustaflavone A isolated from *Selaginella trichoclada* (Selaginellaceae) induced ferroptosis in breast cancer cells MCF-7 with a cytotoxic IC_50_ value of 11.89 µM (doxorubicin 12.62 µM, MTT test) [57]. It (5 and 10 µM) decreased the expression of E3 ubiquitin ligase Nedd4 (neuronal precursor cell-expressed developmentally downregulated 4), thereby reducing the ubiquitination and proteasomal degradation of VDAC2 proteins. In response, VDAC2 protein expression was enhanced, leading to lipid peroxidation and ROS production in mitochondria and MCF-7 cell death. After the addition of the ferroptosis inhibitors, the antioxidant ferrostatin-1, or the iron chelator deferoxamine, the MCF-7 viability was significantly increased. Recently, by RNA sequencing and KEGG functional enrichment analysis, robustaflavone A was shown to reduce the expression of ferroptosis-related genes including *ACSL4*, *NOXO1*, *NOXA1*, *ACSL5*, *STEAP3*, *LPCAT3*, *ATG7,* and *TP53* in MCF-7 cells [58].

Another biflavonoid of apigenin, widely distributed in the *Selaginella* species, amentoflavone was reported to have a multitarget anticancer ability. This polyphenol (10 and 20 μM) was found to suppress growth and induce death of glioblastoma cells U251 and U373 by triggering ferroptosis in an autophagy-dependent manner [59,60]. Its inhibitory effect on cell proliferation was suppressed by deferoxamine and ferrostatin-1 as well as by the upregulation of FTH1. In cells U251 and U373, amentoflavone increased intracellular levels of iron, malondialdehyde (MDA), and lipid ROS and decreased GSH level and mitochondrial membrane potential. Selectively, in glioma cells, and not in normal human astrocytes, amentoflavone induced ferroptosis by modulating iron homeostasis through suppression of FTH1 levels. It induced autophagy via the AMPK/mTOR/p70S6K pathway, which resulted in the downregulation of FTH1 expression. Its effect on inhibition of tumor growth by inducing autophagy-dependent ferroptosis was additionally demonstrated in vivo in a xenograft mouse model.

Erianin is a natural polyphenol found in *Dendrobium chrysotoxum Lindl* (Orchidaceae), and it has anticancer activity against various cancers—osteosarcoma, nasopharyngeal carcinoma, bladder, and lung cancer. Erianin inhibited the proliferation and metastasis of lung cancer via calcium/calmodulin-dependent ferroptosis in vitro and in vivo [61]. In lung cancer cells H460 and H1299, erianin (at concentrations ranging from 12.5 to 100 nM) induced G2/M-phase arrest, ROS accumulation, lipid peroxidation, GSH depletion, and downregulation of the expression of the negative regulatory proteins for ferroptosis, GPX4, CHAC2, SLC40A1, SLC7A11, and glutaminase. The ferroptosis inhibitors ferrostatin-1 and liproxstatin-1, but not the pan-caspase inhibitor Z-VAD-FMK, the potent inhibitor of autophagy chloroquine, or the potent inhibitor of necroptosis necrostatin-1, rescued cells from erianin-induced cell death. Calcium/calmodulin signaling is a critical mediator of erianin-induced ferroptosis. The blockade of this signaling with ruthenium red and antagonist calmidazolium, significantly rescued cell death induced by erianin treatment by suppressing ferroptosis. Inhibition of calcium/calmodulin signaling significantly reduced the expression of transferrin and increased the expression of GPX4 and SLC7A11. Ferroptosis was also found to contribute largely to the erianin-induced cell death of bladder cancer in vitro and in vivo [62]. Erianin (100 µg/mL, i.e., 314 μM) suppressed the growth of bladder cancer cell lines KU-19–19 and RT4. It induced G2/M-phase arrest, ROS, and MDA accumulation, GSH depletion, and downregulation of ferroptosis-related proteins FTH1, GPX4, HO-1, glutaminase, and X_c_^‾^/SLC7A11 and inactivated NRF2. The compound *tert*-butylhydroquinone, an NRF2 activator, suppressed erianin-induced ferroptosis, whereas NRF2 inhibition by shRNA augmented the ferroptosis response induced by erianin treatment.

Epunctanone, a polyprenylated benzophenone isolated from two African plants *Garcinia epunctata* (Clusiaceae) and *Ptycholobium contortum* (Fabaceae)*,* has been identified as a promising cytotoxic molecule against nine cancer cell lines including multidrug-resistant ones [63]. It was shown to induce ferroptosis in addition to apoptosis in leukemia cells CCRF-CEM (at a concentration of 11.8 μM).

Piperlongumine, an electrophilic, natural compound isolated from the long pepper *Piper longum* L. (Piperaceae), was selectively toxic to cancer cells in vitro and in vivo [64,65]. It induced the death of human pancreatic cancer cells PANC-1, in part by ferroptosis [66]. The combined treatment of piperlongumine (5–10 µM) with the plant growth regulator cotylenin A (24.1 µM) and/or the clinically approved ferroptosis inducer sulfasalazine (200 µM) enhanced PANC-1 cell death. Its cancer-cell-killing activity was inhibited by the antioxidant *N*-acetylcysteine (3 mM) and the ferroptosis inhibitors ferrostatin-1 (1 µM) and liproxstatin-1 (1 µM), as well as the iron chelator deferoxamine (200 µM), but not by the apoptosis inhibitor Z-VAD-FMK or the necrosis inhibitor necrostatin-1. Piperlongumine in the form of a nanosystem was found to induce the non-apoptotic cell death of mouse breast cancer cell line TH1 via ferroptosis and pyroptosis (an inherently inflammatory kind of programmed cell death activated by caspases 1, 4, and 5). The nanosystem Tf-LipoMof@PL consists of piperlongumine (PL) encapsulated in an iron-containing metal–organic framework (MOF) coated with a transferrin-decorated, pH-sensitive lipid layer (Tf-Lipo). In this multifunctional nanosystem, piperlongumine contributed to ferroptotic cell death by providing H_2_O_2_ to increase ROS generation through the Fenton reaction [64]. Transferrin facilitated the accumulation of intracellular iron levels, while a pH-sensitive DOPE (dioleoylphosphatidylethanolamine) lipid layer enhanced cellular uptake, prevented early drug leakage, and enabled pH-responsive piperlongumine release in response to low pH at the tumor site. The anticancer effect of the dual-inductive nanosystem was demonstrated in vivo on the xenograft mice model.

The anticancer effect of classical chemotherapeutics, targeted drugs, or radiotherapy can be enhanced by combination with ferroptosis inducers [47]. Gallic acid (3,4,5-trihydroxy benzoic acid, GA) is a phenolic compound with anticancer and antioxidant properties [67]. It was found to cause the death of cervical cancer cells HeLa by early-stage ferroptosis via inhibition of GPX4 activity, mid-stage apoptosis, and late-stage necroptosis at 50 μg/mL concentration (i.e., 294 µM) [68,69]. In another study, while low-level laser irradiation (red) was unable to cause death in breast (MDA-MB-231) and melanoma (A375) cancer cell lines, the pre-irradiation followed by treatment with gallic acid (IC_50_ of 25 and 50 μg/mL, i.e., 147 µM and 294 µM, respectively) reduced the cancer cell survival significantly more than gallic acid alone [68]. The viability of the human skin fibroblasts was not altered, and the effect was greater than that caused by the first treatment with gallic acid followed by low-level laser irradiation. Irradiation of the cells promoted penetration of gallic acid and caused (in addition to apoptosis) ferroptosis via decreasing GPX4 activity and increasing lipid peroxidation. The biomimetic nanoreactor was composed of 4,4′-azonzenecarboxylic acid (Azo)–BSA functionalized hybrid zeolitic imidazolate framework (ZIF), which encapsulated Fe(III)–gallic acid and glucose oxidase (GOx, for sustained oxygen consumption resulting in hypoxia microenvironment) [70]. This Fe(III) –GA/GOx@ZIF–Azo nanoplatform was applied to the MCF-7 breast cell line. It enabled hypoxia-activated positive feedback of cellular uptake and more efficient ferroptotic therapy. The Fenton reaction was accelerated not only by the sustained supply of Fe^2+^ and H_2_O_2_ but also by the low pH and photothermal stimulation of the reduction of Fe^3+^ to Fe^2+^ ions. Gallic acid has also been used for the synthesis of nanoprobes applicable for magnetic resonance imaging and photothermal cancer therapy [71]. The nanoprobes were constructed using luminescence nanoparticles (UCNP) as the core and the Fe^3+^/gallic acid complex as the shell, allowing the release of Fe^3+^ ions in the tumor microenvironment in response to the slightly acidic pH. UCNP@GA-FeIII probes acquire specific tumor-targeting ability by absorbing the unsaturated transferrin from serum that recognizes the overexpressed TfR1 on the surface of various solid, malignant tumor cells, including colorectal cancer cells LS180.

### 5.2. Ferroptosis Inhibitors

Ferroptosis inhibitors can act as GPX4 activators, free radical scavengers, iron chelators, and/or NRF2 activators. The most commonly used ferroptosis inhibitors for in vitro experiments, ferrostatin-1 and liproxstatin-1, act as antioxidants. Many polyphenols are well characterized as radical scavengers, iron chelators, and/or NRF2 activators [51,72], which contributes to their suppressive effect on ferroptosis. The mechanism of their antioxidant effects depends on the number and position of hydroxy groups on the core benzo-γ-pyrone fragment, as well as on the dose and cell type [7].

The food flavonoid apigenin (20 µM) inhibited ferroptosis induced by kainic acid in human neuroblastoma cells SH-SY5Y [73]. Apigenin also rescued mouse brain in vivo from myeloperoxidase (MPO)-mediated oxidative stress. Kainic-acid-induced upregulation of MPO, and, thus, HClO generation, was accompanied by reduced activities of SIRT1 and GPX4, while apigenin treatment decreased expression of MPO and upregulated expression of SIRT1 and the intracellular antioxidants GPX4, TrxR (thioredoxin reductase), and GSH. Conversely, in multiple myeloma cell line NCl-H929, apigenin (5–40 µM) induced cell death by ferroptosis even in the presence of ferrostatin-1 and deferoxamine [74]. Chloroform extract from *Fumaria officinalis* (Fumariaceae), which contains two flavonoids, apigenin and isoquercetin, in addition to isoquinoline alkaloids, also stimulated iron-dependent death in the cell line NCI-H929 [75]. Apigenin also induced the death of lung cancer cells A549 partly through ferroptosis (in addition to apoptosis) [76]. The ferroptotic effect of apigenin (API) on A549 cells was synergistically enhanced by its incorporation into magnetic iron oxide nanoparticles modified on the surface with polysaccharide hyaluronic acid Fe_2_O_3_/Fe_3_O_4_@mSiO_2_-HA. Such a nanocomposite enabled the sustained release of poorly water-soluble apigenin and the specific targeting of cancer cells with expressed channel protein CD44 on the cell membrane. The API-Fe_2_O_3_/Fe_3_O_4_@mSiO_2_-HA nanosystem was found to significantly increase ROS and cell lipid peroxidation levels, as well as downregulate GPX4 and FTH1 in A549 cells, compared to pure apigenin.

**Figure 4:**
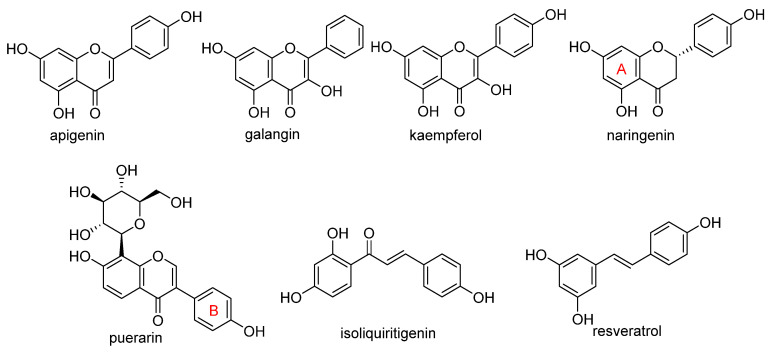
Flavonoids and resveratrol with resorcinol and/or *para*-OH phenyl moieties showing antiferroptotic activity.

Two other 3-hydroxy flavones, or so-called flavonols, galangin and kaempferol (Figure 4), protect neurons from ferroptosis. Galangin, a flavonol from the Chinese medicinal herb *Alpinia officinarum* (Zingiberaceae), protected hippocampal neurons in gerbils after ischemia reperfusion by inhibiting ferroptosis via activation of the SLC7A11/GPX4 axis. A deficiency of GSH indirectly caused suppression of GPX4 activity [77]. Kaempferol has been used to treat neuronal cells after oxygen-glucose deprivation/reoxygenation associated with ischemic stroke. It protected cells by activation of the NRF2/SLC7A11/GPX4 signaling pathway [78].

Naringenin, a flavanone from fruits and herbs, was studied in cardiomyocytes H9C2 [79]. Hypoxia reperfusion induced with erastin in cardiomyocytes was alleviated by naringenin, as it increased the expression of proteins NRF2, SLC7A11, GPX4, FTH1, and the iron export protein ferroportin 1 (FPN1) and decreased the expression of NADPH oxidase NOX1. Iron uptake mediated the activation of NOX1 signaling, which induced the release of ROS and mitochondrial damage. All four polyphenols shared resorcinol moiety in ring A (Figure 4) and suppressed ferroptosis through regulation of the NRF2/SLC7A11/GPX4 axis.

Another polyphenolic antioxidant with a *para*-OH group in B-ring puerarin, 8C-glucoside of isoflavone daidzein (Figure 4), was shown to inhibit ferroptosis and lipopolysaccharides (LPS)-induced inflammatory response in A549 cells. Total iron and divalent iron levels, lipid peroxidation, and the NOX1 expression were decreased significantly upon addition of puerarin in LPS-modified A549 cells. In contrast, the expression of SLC7A11, GPX4, and FTH1 was increased [80].

There are plenty of structurally diverse polyphenols containing catechol (1,2-dihydroxy benzene) or galloyl (1,2,3-trihydroxy benzene) moieties, which are responsible for their strong antioxidant activity. In addition to their high potential to directly scavenge free radicals, they can form complexes with Fe^2+^ ions, which can further stimulate the oxidation of Fe^2+^ to Fe^3+^ and prevent the recycling of Fe^2+^ ions [81].

**Figure 5:**
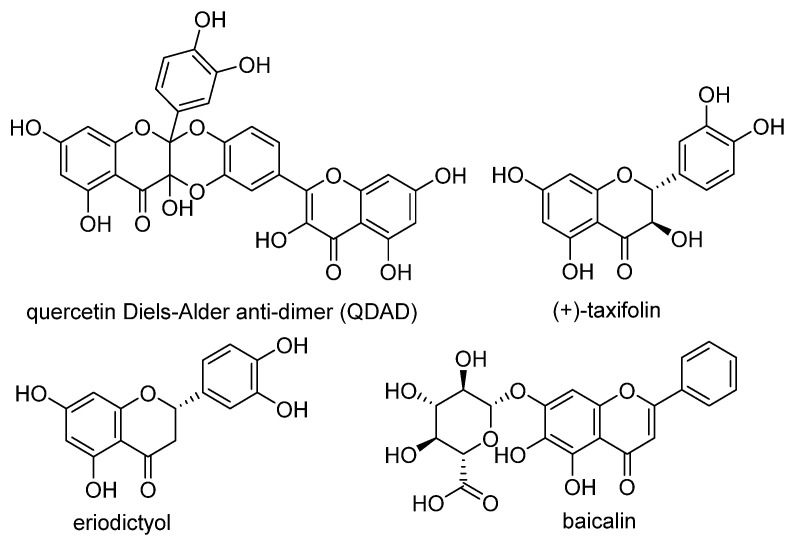
Antiferroptotic flavonoids with catecholic moiety not included in Table 2.

Quercetin, named after *Quercus* sp. (oak tree) [82], is a catecholic flavonol. Its antioxidant and chelating effects, as well as its anticancer activity, have been reviewed many times [6,83,84,85,86]. Its metal-chelating activity has been linked to antioxidant activity, and its metal complexes also exert antioxidant activity [82,87]. Quercetin could chelate Fe^2+^ ion by catechol functionality at a stoichiometric ratio of 1:2 metal/ligand and attenuated lipid peroxidation, as well as protein oxidation, in the liver, kidneys, and hearts of mice overloaded by iron–dextran complex [88]. Quercetin is more toxic to cancer cells than to normal cells [83]. While quercetin has antioxidant and chemopreventive effects at low concentrations, it exhibits pro-oxidant effects at high doses [86,89]. The oxidized product quercetin quinone is responsible for its pro-oxidant activity, and the pro-oxidant effects of quercetin also depend on the intracellular GSH levels [51]. Quercetin can also exist in plants as quercetin Diels–Alder anti-dimer (QDAD, Figure 5). The antiferroptotic activity of quercetin and its biflavonoid QDAD was investigated in erastin-induced ferroptosis in a bone-marrow-derived mesenchymal stem cell model [90]. Quercetin had better antiferroptotic activity than QDAD, which may be due to the stronger antioxidant action of quercetin. The IC_50_ value for the antioxidant effect against lipid peroxidation for quercetin (2.2 µM) was better than for QDAD (15.5 µM) and referent antioxidants trolox (136 µM) and ascorbic acid (3.0 µM). Quercetin alleviated ferroptosis in pancreatic β-cells in diabetic mice in vivo [91]. It also inhibited ferroptosis and subsequent inflammation in renal proximal tubular epithelial cells, which contributed to the mitigation of acute kidney injury in vivo [92]. Quercetin reduced MDA and lipid ROS levels and increased GSH level. It was found to inhibit ferroptosis by blocking the expression of activation transcription factor 3 (ATF3), the activation of which plays an important role in cell ferroptosis. It did not affect the ATF3 level in normal mice.

Taxifolin (Figure 5) is a dihydrogenated quercetin derivative belonging to the flavanonol subclass of flavonoids. Its synthetic 7-*O*-esters with cinnamic and ferulic acids (Table 2) showed antiferroptotic activity in the mouse hippocampal neuron cell model HT22, in a way that was different from parent taxifolin and phenolic acids [93]. The HT22 cell line represents immortalized mouse hippocampal neuronal cells that do not express cholinergic and glutamate receptors such as mature hippocampal neurons in vivo. They are commonly used in in vitro studies of the neuronal differentiation and neurotoxicity implicated in brain injuries or neurological diseases.

Taxifolin’s analog, without the group 3-OH and with the methylated 7-OH group, the flavanone sterubin from Yerba santa (*Eriodictyon californicum*, Boraginaceae), was studied in association with neuroprotective compounds that can be used against Alzheimer’s disease [94]. It was also found to have dose-dependent protection against ferroptosis inducers erastin and RSL3 in hippocampal cells HT22. It has a chelating ability that has been determined in a ferrozine-based assay.

The demethylated derivative of sterubin, eriodictyol (Figure 5), found in the peel of citrus fruits and some Chinese herbal medicines, also showed an anti-Alzheimer’s-disease effect through antiferroptotic activity in vitro in HT22 cells and in vivo in the APP/PS1 mouse model of Alzheimer’s disease [95]. Treatment with eriodictyol (50 mg/kg) inhibited ferroptosis in the brains of APP/PS1 mice by reduction of the levels of Fe^2+^ ions and total iron, as well as the reduction of the expression levels of TfR1 and FTH1, and increase of the GPX4 and vitamin D receptor (VDR) levels in the cortex and hippocampus of APP/PS1 mice. The iron export protein FPN1 was also upregulated upon eriodictyol treatment. This suggests that eriodictyol might maintain iron balance in cells by reducing iron intake and increasing iron output. The mechanism of antiferroptotic action investigated in HT22 cells was shown to be related to the activation of the NRF2/heme oxygenase-1 (HO-1) signaling pathway mediated by VDR.

By screening a natural product library, a flavon baicalein with the galloyl ring A, was found to be a ferroptosis inhibitor. It markedly inhibited erastin-induced ferroptosis in pancreatic cancer cells compared to known ferroptosis inhibitors. It limited erastin-induced Fe^2+^ iron accumulation, GSH depletion, and lipid peroxidation by suppressing erastin-mediated degradation of GPX4 [96]. Another mechanism that may contribute to the antiferroptotic effect of baicalein is the significant and selective inhibition of 12/15-lipoxygenase (12/15-LOX), which was demonstrated in an experiment with RSL3-stimulated lipid peroxidation in acute lymphoblastic leukemia (ALL) model cell lines Molt-4 and Jurkat [97]. In a study focused on Alzheimer’s disease, baicalein decreased ferroptosis markers lipid ROS, 4-hydroxynonenal, and COX2 (cyclooxygenase-2) and inhibited the expression of 12/15-LOX in a HT22 cell model of iron-induced injury, which was also validated in a mouse model of posttraumatic epilepsy [98]. The antiferroptotic effect of baicalein was also confirmed in melanocytes in vitiligo. Baicalein upregulated GPX4 and reduced TfR1 levels in melanocytes treated with RSL3 and ferric ammonium citrate [99]. 7-*O*-glycoside baicalin (Figure 5), found in the roots of the traditional Chinese medicinal plant *Scutellaria baicalensis* (Lamiaceae), suppressed autophagy-dependent ferroptosis in a study which focused on early brain injury following subarachnoid hemorrhage. The study was based on in vitro and in vivo models, and the evaluated parameters were Fe^2+^, MDA, ROS, and GSH levels [100]. However, in contrast, baicalin exerted anticancer activity by triggering FTH1-dependent ferroptosis in bladder cancer cells in vitro (in cell lines 5637 (50–60 μg/mL, i.e., 112–134 µM) and KU-19-19 (100–120 μg/mL, i.e., 224–269 µM)) and in vivo (in mice, 200 mg/kg), accompanied by intracellular ROS and iron accumulation [101]. The ferroptosis inhibitor deferoxamine rescued baicalin-induced cell death in both 5637 and KU-19-19 cell lines.

A change in iron concentration may influence the dominant mechanism of action of polyphenols. While an excess of iron does not influence the beneficial effects of flavanone sterubin, this is not the case with flavonol fisetin. Differently to Cu^2+^ ions, Fe^2+^ ions have a significant influence on its anti-inflammatory effect [102]. The antioxidant activity of fisetin, suppression of ROS production, and maintenance of GSH levels are altered differently by metals. Fisetin (5 µM) has been tested in several assays in association with its neuroprotective and anti-inflammatory activities. It has direct antioxidant activity and can also chelate Fe^2+^ ions. Fisetin induced NRF2 in hippocampal HT22 cells and microglial BV-2 cells, but not in the presence of Fe^2+^. Iron ions blocked NRF2 induction by fisetin in cells of both types. Fisetin additionally reduced glutamate-induced ROS production, but the presence of Fe^2+^ also blocked this effect. In HT22 cells, fisetin completely blocked the ROS production induced by RSL3. While ROS production was not significantly increased by Fe^2+^ ions, it was greatly potentiated by the combination of RSL3 and Fe^2+^ ions with fisetin. This was attributed to the ability of iron to oxidize fisetin and, thereby, change fisetin’s effect on the induction of antioxidant transcription factor NRF2. The oxidized form of fisetin acts as a strong pro-oxidant. In contrast, although sterubin also binds iron, the metal does not affect the ability of sterubin to induce NRF2.

Gossypitrin is a hydrophilic 7-*O*-glucoside of flavonol gossypetin, isolated from *Talipariti elatum* Sw. (Majagua azul from the Malvaceae family), which has antioxidant activity [103]. It was tested on iron-induced oxidative damage in HT22 cells and mitochondria isolated from rat brains [104]. It was able to rescue HT22 cells from damage induced by 100 µM Fe(II)-citrate, with an EC_50_ of 8.6 µM. The effect was associated with the prevention of iron-induced mitochondrial membrane potential dissipation and ATP depletion. This substance also prevented Fe(II)-citrate-induced mitochondrial lipid peroxidation with an IC_50_ value of 12.45 µM, which was about nine times more efficient than the prevention of *tert*-butylhydroperoxide-induced peroxidation. It also decreased Fe^2+^ concentration with time, while increasing the O_2_ consumption rate and impairing Fe^3+^ reduction by ascorbate. Gossypitrin forms a complex with ferrous Fe^2+^ ions in a 2:1 ratio, accelerates its oxidation to a more stable complex with iron in the ferric Fe^3+^ state, and, thus, impedes iron recycling back to the pro-oxidant Fe^2+^ state required for the ROS production and, thus, suppresses the propagation phase of lipid peroxidation.

**Figure 6:**
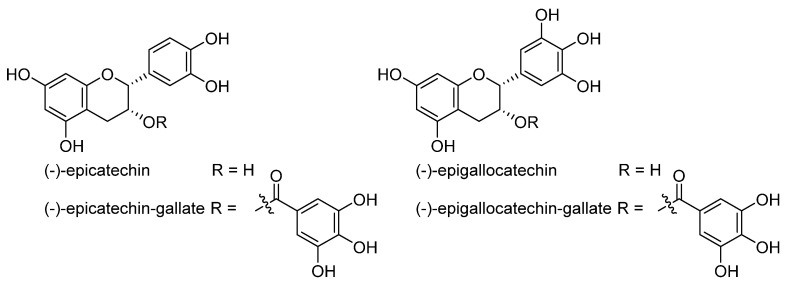
Structure of catechin derivatives with observed antiferroptotic activity.

The flavonoid group also includes catechins (Figure 6), which belong to the subgroup of flavan-3-ols. These compounds are present in fruits, vegetables, various beverages, wine, juice, cocoa, and chocolate. They are known to be favorable components of green tea [105]. They have cardiac and neurological beneficial effects [106]. The anticancer activities of catechins through various mechanisms have been summarized, and controlled cell death has also been implicated in the mechanisms [107]. (−)-Epigallocatechin (EGC) and (−)-epigallocatechin gallate (EGCG), as well as other catechins, are potent scavengers of superoxide radicals, but they may also act as pro-oxidants [7,108]. The ability to chelate Fe^3+^ decreases in the order EGCG > epicatechin gallate (ECG) > EGC > (−)-epicatechin [106]. Brain-permeable (−)-epicatechin (15 mg/kg) reduced lesion volume and ameliorated neurologic deficits in a collagenase model of intracerebral hemorrhage in mice. It downregulated ferroptosis-related gene expression and acted through NRF2-dependent and independent pathways [109]. The neuroprotective function of EGCG was investigated in cerebellar granule neurons as a simulation of spinal cord injury. EGCG (50 μM) increased the survival rate, inhibited ferroptosis, and upregulated phosphorylation of protein kinase D1 under ferroptotic conditions. The effect was verified in rats [110]. Pretreatment of pancreatic β-cell line MIN6 with EGCG or curcumin (20 μM) inhibited the iron accumulation and ferroptotic cell death triggered by erastin. Both polyphenols attenuated GSH depletion, GPX4 inactivation, and lipid peroxidation [111].

In contrast, EGCG has been used in the preparation of nanocarriers aimed at inducing ferroptosis selectively in cancer cells. Since EGCG can reduce Fe^3+^ to Fe^2+^, it has been used in the preparation of nanoparticles with the trio doxorubicin/Fe^3+^/EGCG, able to induce cancer cell death via ferroptosis and apoptosis. Fe^3+^ ions are released after uptake by cancer cells because cancer cells are acidic and have a high level of glutathione. EGCG is responsible for intracellular iron reduction and the consequent production of hydroxyl radicals through the Fenton reaction and induction of ferroptosis, which enhanced doxorubicin-induced apoptosis in mouse lung carcinoma cell line LL2 [112].

Chalcones (1,3-diphenylprop-2-en-1-ones) are compounds of natural origin serving as starting components for flavonoid biosynthesis in plants [113,114]. Due to the interesting range of biological activities of chalcone derivatives and analogs, the chalcone scaffold is considered to be an important synthetic moiety in medicinal chemistry [115,116]. The antiferroptotic activity of synthetic, hydroxylated chalcones ((2*E*)-3-(3-methylphenyl)-1-(2,3,4-trihydroxyphenyl)prop-2-en-1-one (synthetic chalcone 1 in Table 2), (2*E*)-3-(4-chlorophenyl)-1-(2,3,4-trihydroxyphenyl)prop-2-en-1-one, and (2*E*)-3-(4-methoxyphenyl)-1-(2,3,4-trihydroxyphenyl)prop-2-en-1-one), each containing the galloyl ring A, was tested for lipid peroxidation inhibition in cellular assays. All three compounds were included in the set of inhibitors for chemoinformatic analysis (Table S2). Pretreatment of cells with each of the chalcones (25 μM) was shown to inhibit amyloid-β peptide (Aβ) aggregation in human neuroblastoma SH-SY5Y cells as well as ferroptosis in human embryonic kidney HEK-293 cells. They were able to reduce lipid peroxidation stimulated by RSL3 or erastin in HEK-293 cells with an IC_50_ value of 0.45–1.77 µM and 3.15–3.88 µM, respectively, whereas ECGC did not show any effect up to the concentration of 20 µM [117].

The natural chalcone butein was studied to inhibit ferroptosis, and its antioxidant effect was compared to that of its cyclized product flavanone butin at a dose of 30 μM. Butein inhibited ferroptosis more effectively in erastin-treated, bone-marrow-derived mesenchymal stem cells and showed a stronger antioxidant effect in five different antioxidant assays than butin. The authors concluded that butein exerts an anti-apoptotic effect due to antioxidant action based on the hydrogen-atom transfer pathway. The difference in action between butein and butin was attributed to the decrease in π–π conjugation in butein due to the saturation of the α,β double bond and loss of the 2-hydroxy group upon biocatalytical isomerization [118].

Morachalcone D (Table 2) and morachalcone E are prenylated chalcones isolated from mulberry leaves. In HT22 cells, morachalcone D attenuated erastin-induced ferroptosis with an EC_50_ value of 33.7 ± 0.89 µM. Its effect was compared to quercetin EC_50_ 9.55 ± 0.43 µM. Morachalcone E was only slightly active, which might have been influenced by the elimination of the active hydroxy group and different prenyl pattern. Morachalcone D inhibited the iron accumulation triggered by erastin, which was confirmed by FeRhoNox™-1, an activatable probe detecting labile Fe^2+^ ions in living cells via orange fluorescence. It also upregulated the expression of genes involved in antioxidant defense, including *GPX4*, *CAT*, *SOD2*, *NRF2*, *HMOX1,* and *SLC7A11*, in erastin-treated HT22 cells in a dose-dependent manner [119].

The antiferroptotic effect of isoliquiritigenin (Figure 4), a component of licorice root, was detected in human kidney epithelial tubular cell line HK2. Pretreatment of the cells with this chalcone inhibited Fe^2+^ ions accumulation and lipid peroxidation in LPS-stimulated HK2 cells. Isoliquritigenin increased the expression of GPX4 and the chain subunit of the cystine/glutamate transporter SLC7A11 and attenuated mitochondria injury in renal tubular following LPS injection in mice [120].

In a study on constituents of traditional Chinese medicine, coumarins and coumestans were isolated from the fruits of *Psoralea corylifolia* (syn. *Cullen corylifolium*, Fabaceae). Their activity was explored in erastin-exposed HT22 cells. Among the isolated phytochemicals, the coumestan psoralidin was the most active one, with an IC_50_ of 5.21 µM (for comparison, IC_50_ for the standard compound ferrostatin-1 was 0.45 µM). The coumarins psoralen and isopsoralen showed no significant activity [121].

The stilbenoid resveratrol protects cells against oxidative agents at low doses but can promote the production of ROS at high concentrations. It stimulates the KEAP1/NRF2 pathway by activating NRF2 and increasing its expression [122,123]. Its precise mechanism of action is the modification of the amino acid residue Cys151 in KEAP1, which causes newly formed NRF2 to escape from ubiquitination [124]. In mouse pancreatic β cells MIN6, resveratrol inhibited ferroptosis induced by acrolein, a food and environmental pollutant and a risk factor for diabetes. The inhibition was determined by analysis of the biomolecules associated with ferroptosis GPX4, COX2, ACSL4, MDA, GSH, and 5-hydroxyeicosatetraenoic acid (HETE) [125]. The ferroptosis inhibitory effect of resveratrol was also observed in an oxygen-glucose deprivation/reoxygenation model of myocardial ischemia–reperfusion injury in H9C2 cells. Resveratrol (10 μM) alleviated induced oxidative stress and inhibited ferroptosis. It decreased TfR1 expression and increased the expressions of FTH1 and GPX4. It was found to inhibit ferroptosis via the regulation of ubiquity-specific peptidase 19 (USP19)-Beclin1-induced autophagy. The attenuating effect on ferroptosis was also confirmed in vivo in rats [126]. Resveratrol is also known for its neuroprotective effects, but its poor oral bioavailability limits its clinical application. The poor bioavailability of resveratrol has been improved by incorporation into MPEG-PLGA nanoparticles. MPEG-PLGA nanoparticles containing resveratrol accumulated in the endoplasmic reticulum and lysosomes in Madin–Darby canine kidney (MDCK) cells and passed across physiological barriers in a zebrafish model. They inhibited erastin-induced ferroptosis in mouse hippocampal HT22 cells and an intracerebral hemorrhage injury mouse model [127].

However, in head and neck cancer cells HN3 and HN4, resveratrol (20 μM) increased ferroptosis when used in combination with RSL3. Resveratrol is a histone deacetylase SIRT1 inducer. The pharmacological activation of SIRT1 and associated epithelial–mesenchymal transition (EMT) epigenetic reprogramming induced by resveratrol lead to increased sensitivity to the ferroptosis inducer RSL3. The cell viability was significantly decreased after the addition of resveratrol due to a weakened antioxidant system [128].

Another non-flavonoid, polyphenol curcumin (Table 2), is the main component of turmeric, a dietary spice extracted from the root of *Curcuma longa* (Zingiberaceae). This curcuminoid exerts plenty of biological activities, including anticancer and neuroprotective ones. It possesses strong antioxidant activity at low concentrations, whereas, at higher concentrations, it behaves as a potent pro-oxidant. In association with ferroptosis, iron chelation by curcumin is worth mentioning. Its chelating ability was demonstrated at a concentration of 25 μM in liver cancer cell line Huh7 with iron overload obtained by fe-nitriloacetic acid or ferric ammonium citrate [129]. Curcumin is known to have renoprotective properties. It has been studied in an acute kidney injury model in mice caused by rhabdomyolysis. Pre- and posttreatment with curcumin (at a dose of 1 g/kg intraperitoneally) mediated HO-1 induction to prevent oxidative stress and inflammation in vivo. Analogous to the in vivo results, an in vitro mechanistic study conducted in proximal murine tubular epithelial cells showed that pretreatment with curcumin (10 μM) also reduced TLR4/NF-kB and ERK1/2 activation. Within this study, it was demonstrated that ferroptosis is involved in rhabdomyolysis-associated renal damage [130]. The disadvantages of curcumin, such as its poor water solubility, limited oral bioavailability, and inability to efficiently transit across physiological barriers, can be overcome by its encapsulation into polymer-based nanoparticles (Cur-NPs). Cur-NPs (PEG-PTMC) were shown to attenuate the severity of intracerebral hemorrhage injury in a mice model and to suppress erastin-induced ferroptosis in HT22 cells. The absorption, distribution, and elimination properties of Cur-NPs were explored in vitro in MDCK cells and a zebrafish model and in vivo in the brain and plasma of treated mice. Cur-NPs were accumulated in lysosomes, the endoplasmic reticulum, and mitochondria. In HT22 cells, Cur-NPs inhibited ROS production by regulating the NRF2/HO-1 pathway [131]. Due to the presence of two enone moieties in the structure, it can react with Cys151 in KEAP1 to permit NRF2 dissociation and stabilization [128].

However, in cancer cells, curcumin has been shown to induce ferroptosis. Curcumin has anticancer properties that operate through a variety of mechanisms, including inhibition of cancer cell proliferation, invasion and metastasis, regulation of apoptosis, and autophagy. It has been shown to inhibit glioblastoma, breast, and non-small-cell lung cancer (NSCLC) cells via the regulation of ferroptosis. It induced characteristic changes of ferroptosis in vivo in tumor tissues and in vitro in cancer cell lines. Curcumin significantly triggered the cytological and molecular characteristics of ferroptotic cell death in LLC (Lewis lung cancer)-bearing mice (dose 100 mg/kg/day intraperitoneally) and in A549 and H1299 cells (at a 30 μM dose), including depletion of GSH, lipid peroxidation, and accumulation of ROS and iron. In the tumor tissue of mice, the protein level of ACSL4 was upregulated, while the protein levels of SLC7A11 and GPX4 were significantly downregulated by curcumin [132]. In breast cancer cell lines MCF-7 and MDA-MB-231, curcumin (with cell viability IC_50_ values after 48 h of 41.90 μM and 53.51 μM, respectively) caused marked accumulation of intracellular iron, ROS, lipid peroxides, and MDA, while it downregulated GSH levels significantly. It was found to upregulate a variety of ferroptosis genes related to redox regulation, including HO-1, but to downregulate the expression of GPX4 [133]. Curcumin also induced ferroptosis in clear-cell renal cell carcinoma (ccRCC) cells resistant to sunitinib, a tyrosine kinase inhibitor which blocks angiogenesis. Curcumin reversed resistance and enhanced the sensitivity of 786-O-DR (drug-resistant) cells to sunitinib. It reduced the iron content, upregulated the expression of the *ADAMTS18* gene, and significantly reduced expression levels of the ferritin autophagic cargo receptor NCOA4 (nuclear receptor coactivator 4) and the proteins FTH1 and p53 in the cells [134].

## 6. Data Set and Methods

The sets of diverse inducers and inhibitors of ferroptosis were collected from the literature. The collected ferroptosis modulators were compared mutually and with drugs in terms of their structural and physicochemical/drug-likeness properties. The set of 1390 approved drugs was downloaded from the open-access drug discovery resource ChEMBL (https://www.ebi.ac.uk/chembl, 29 August 2021) [135]. The Anatomical Therapeutic Chemical (ATC) first-level categories of drugs were collected from databases ChEMBL and KEGG [136].

Ferroptosis modulators and drugs are represented with SMILES and corresponding MACCS keys calculated by the R package *rcdk* [137]. The physicochemical molecular features important for biological activities were calculated by the programs ADMET Predictor™ 10.0 (Simulations Plus Inc., Lancaster, CA, USA) (43 descriptors) [18] and DataWarrior (32 features) [138]. The ionization state of molecules was estimated by the program ADMET Predictor™ (Supplementary Table S1).

Structural similarity of the compounds was estimated by applying clustering in terms of MACCS fingerprints with Jaccard distance as a dissimilarity measure and principal component analysis (PCA). The Jaccard distance equals the difference 1−Tanimoto coefficient (TC). TC is the ratio of the number of common features to the number of different features present in two compared molecules. The clustering and PCA were performed by the R functions *hclust* using the complete linkage method and princomp, respectively [137]. The biplot visualization was carried out with the R package factoextra.

The chemical classes of ferroptosis modulators were determined by the ClassyFire algorithm [42].

All chemical structures have been drawn using the program CS ChemDraw Professional version 20.0.0.41 (PerkinElmer, Waltham, MA, USA).

Information on the ferroptosis-related activities of the selected natural and semisynthetic derivatives was gathered through a literature search.

## 7. Conclusions

**Figure 7:**
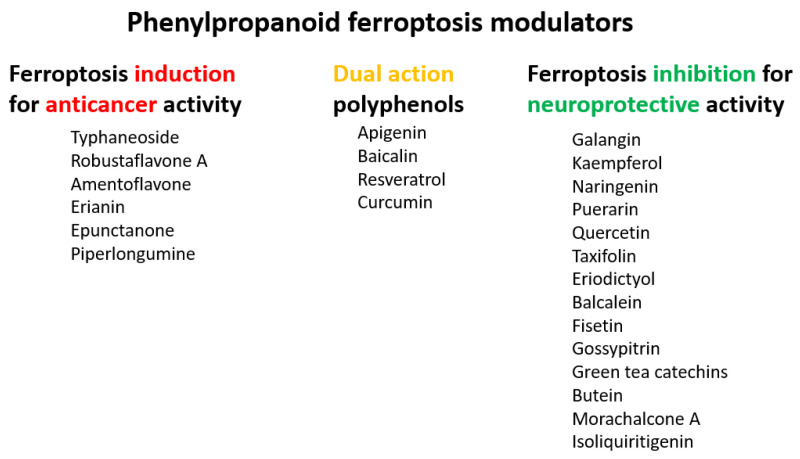
Neuroprotective and anticancer activities of plant ferroptosis modulators from phenylpropanoid biochemical origin and represented in the review.

In the review, we aimed to present the results of our in silico analysis of the collected structurally diverse representatives of ferroptosis modulators in terms of their structural and physicochemical/drug-likeness properties and to summarize in vitro and in vivo results observed mainly for the large subgroup of natural ferroptosis modulators plant (poly)phenols, primarily phenylpropanoids (Figure 7). Most polyphenols (at the micromolar range of concentrations) have antiferroptotic activity, which may contribute to their neuroprotective capacity. The efficient anticancer activity of typhaneoside, robustaflavone A, amentoflavone, and erianin (at μM levels) in vitro and in vivo is ascribed partly to their capacity to induce ferroptosis. However, the effects of polyphenols considerably depend upon their (micro)environment, for example, on the amount and type of free iron. Some polyphenols (apigenin, baicalin, resveratrol, curcumin) can have an inducing or inhibitory effect on ferroptosis depending on the cell type or composition of multifunctional nanoformulation. Such a dependence enables the construction of different kinds of multifunctional, nanoformulated drug delivery systems of active pharmacological ingredients, including polyphenols, which allow selective and specific induction of ferroptosis in pathological cancer cells.

In addition, the performed in silico comparison with approved drugs placed (poly)phenols in the same chemical space as around two-thirds of the drugs from the ATC groups D (dermatologicals), G (genito-urinary system and sex hormones), and H (systemic hormonal preparations, excluding sex hormones and insulins). This observation is in accordance with already performed studies on the anticancer activities of polyphenols on pancreatic, prostate, breast, bladder, and renal cancers and melanoma.

Our results can be used to quickly gain insight into the chemical scaffolds and druglike properties of ferroptosis inducers and inhibitors and to motivate relatively new, targeted use of polyphenols.

## Figures and Tables

**Table 1:** Ferroptosis inducer representatives—chemical structures with the main mode of action ^1^ and predicted lipophilicity coefficient and potential for crossing the blood-to-brain barrier (BBB) by the program ADMET Predictor™ [18].

Inducer Name	2D Structure	Mode of Action	log*P*	BBB
(1*S*,3*R*)-RSL3	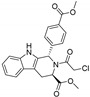	GPX4 ↓	3.6	low
2-phenethylisothiocyanate	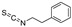	GPX4 ↓	3.4	high
gallic acid	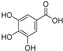	GPX4 ↓	0.7	low
FIN56	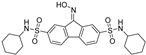	GPX4 ↓	4.7	low
albiziabioside A	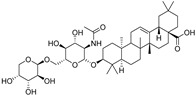	GPX4 ↓	4.5	low
dihydroisotanshinone I	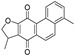	GPX4 ↓	3.7	high
withaferin A	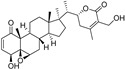	GPX4 ↓	3.2	high
ML162	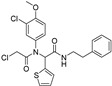	GPX4 ↓	4.4	low
altretamine	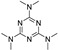	GPX4 ↓	2.5	high
typhaneoside	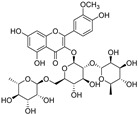	GPX4 ↓	−0.8	low
artemisinin	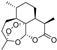	iron ↑; GPX4 ↓	2.4	high
dihydroartemisinin	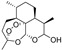	iron ↑; GPX4 ↓	2.2	high
artesunate	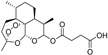	iron ↑	2.3	low
ruscogenin	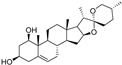	iron ↑	4.5	high
salinomycin	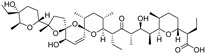	iron ↑	5.4	low
amentoflavone	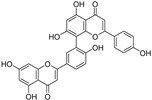	iron ↑	4.5	low
BSO	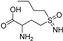	ROS ↑	−2.1	low
FINO2	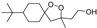	ROS ↑	4.0	high
epunctanone	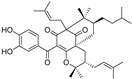	ROS ↑	7.7	low
piperlongumine	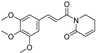	ROS ↑	1.9	high
robustaflavone A	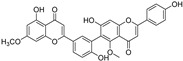	ROS ↑	4.6	low
sorafenib	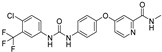	X_c_^‾^ system ↓	5.1	low
erastin	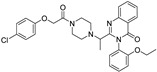	X_c_^‾^ system ↓	3.8	low
sulfasalazine	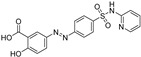	X_c_^‾^ system ↓	3.1	low
ferroptocide	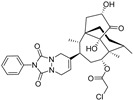	Other	2.1	low
maneb	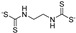	other	0.2	low
gambogic acid	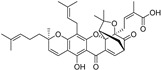	other	7.4	low
paraquat	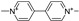	other	−6.3	low
soyauxinium	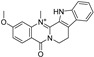	other	0.4	high
erianin	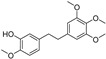	other	3.4	high

^1^ Mode of action: GPX4 ↓ inhibition of GPX4; iron ↑/ROS ↑ intracellular accumulation of iron/ROS; other mechanisms.

**Table 2:** Ferroptosis inhibitor representatives—chemical structures with the main mode of action ^1^ and predicted lipophilicity coefficient and potential for crossing the BBB.

Inhibitor Name	Structure	Mode of Action	log*P*	BBB
butylatedhydroxytoluene	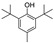	antioxidative	5.5	high
ferrostatin-1	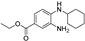	antioxidative	3.7	high
α-tocopherol	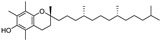	antioxidative	11.5	high
β-carotene	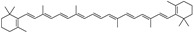	antioxidative	11.6	high
glutathione	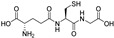	antioxidative	−3.4	low
*N*-acetylcysteine	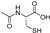	antioxidative	−0.6	low
ascorbic acid	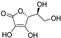	antioxidative	−1.6	low
edaravone	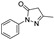	antioxidative	1.3	high
GSK2334470	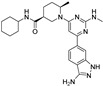	antioxidative	4.3	low
liproxstatin-1	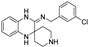	antioxidative	3.3	high
trolox	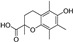	antioxidative	2.9	low
α-lipoic acid	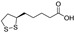	antioxidative; NRF2 ↑	2.7	low
7-*O*-cinnamoyltaxifolin	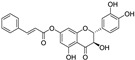	antioxidative	3.7	low
7-*O*-feruloyltaxifolin	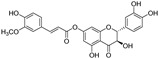	antioxidative	3.1	low
butein	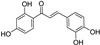	antioxidative	2.8	low
butin	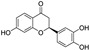	antioxidative	1.9	low
isorhapontigenin	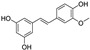	antioxidative	3.0	high
morachalcone D	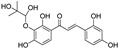	antioxidative	1.7	low
piceatannol-3′-*O*-glucoside	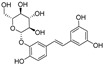	antioxidative	0.4	low
rhapontigenin	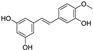	antioxidative	3.1	low
rhapontin	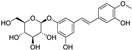	antioxidative	0.6	low
synthetic chalcone 1 ^2^	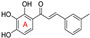	antioxidative	3.8	low
Baicalein ^2^	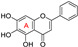	antioxidative; 15-LOX ↓	3.0	low
dopamine	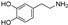	GPX4 ↑	−0.3	low
galangin	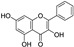	GPX4 ↑	2.7	low
apigenin	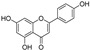	GPX4 ↑	2.9	low
silibinin	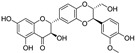	iron chelation	1.8	low
deferoxamine	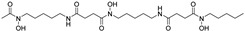	iron chelation	−1.3	low
phytic acid	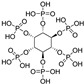	iron chelation	−11.2	high
8-hydroxyquinoline	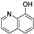	iron chelation	2.1	high
ciclopirox olamine	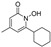	iron chelation	2.5	high
deferasirox	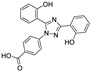	iron chelation	3.8	low
deferiprone	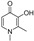	iron chelation	−0.6	high
maltol	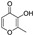	iron chelation	0.0	high
quercetin	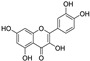	iron chelation	2.0	low
curcumin	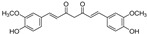	iron chelation	3.0	high
EGCG	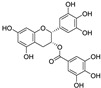	iron chelation	2.2	low
ellagic acid	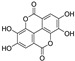	iron chelation	1.9	low
gossypitrin	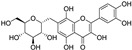	iron chelation	−0.7	low
sterubin	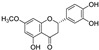	iron chelation	2.0	low
fisetin	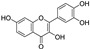	iron chelation; NRF2 ↑	2.2	low
carnosic acid	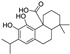	NRF2 ↑	4.5	low
melatonin	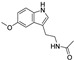	NRF2 ↑	1.7	high
zileuton	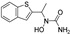	5-LOX ↓	1.9	high
bakuchiol	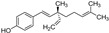	other	5.7	high
psoralidin	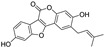	other	4.1	low

^1^ Mode of action: GPX4 ↑/NRF2 ↑ activation of GPX4/NRF2; 5-LOX ↓/15-LOX ↓ inhibition of 5-LOX/15-LOX; antioxidative—radical scavenging; other mechanisms. ^2^ Conventional notation of rings used within text for discussion.

## Data Availability

The data used for analysis in this study are available in the Supplementary Material.

## Supplementary Materials

The following are available online at https://www.mdpi.com/article/10.3390/molecules28020475/s1, Table S1: ClassyFire chemical description, simple molecular descriptors, and physicochemical parameters calculated for the set of 30 ferroptosis inducers, Table S2: ClassyFire chemical description, simple molecular descriptors, and physicochemical parameters calculated for the set of 48 ferroptosis inhibitors.
